# Isotemporal substitution of inactive time with physical activity and time in bed: cross-sectional associations with cardiometabolic health in the PREDIMED-Plus study

**DOI:** 10.1186/s12966-019-0892-4

**Published:** 2019-12-23

**Authors:** Aina M. Galmes-Panades, Veronica Varela-Mato, Jadwiga Konieczna, Julia Wärnberg, Miguel Ángel Martínez-González, Jordi Salas-Salvadó, Dolores Corella, Helmut Schröder, Jesús Vioque, Ángel M. Alonso-Gómez, J. Alfredo Martínez, Luís Serra-Majem, Ramon Estruch, Francisco J. Tinahones, José Lapetra, Xavier Pintó, Josep A. Tur, Antonio Garcia-Rios, Blanca Riquelme-Gallego, José Juan Gaforio, Pilar Matía-Martín, Lidia Daimiel, Rafael Manuel Micó Pérez, Josep Vidal, Clotilde Vázquez, Emilio Ros, Ana Garcia-Arellano, Andrés Díaz-López, Eva M. Asensio, Olga Castañer, Francisca Fiol, Luis Alfredo Mira-Castejón, Anai Moreno Rodríguez, Juan Carlos Benavente- Marín, Itziar Abete, Laura Tomaino, Rosa Casas, F. Javier Barón López, José Carlos Fernández-García, José Manuel Santos-Lozano, Ana Galera, Catalina M. Mascaró, Cristina Razquin, Christopher Papandreou, Olga Portoles, Karla Alejandra Pérez-Vega, Miguel Fiol, Laura Compañ-Gabucio, Jessica Vaquero-Luna, Miguel Ruiz-Canela, Nerea Becerra-Tomás, Montserrat Fitó, Dora Romaguera

**Affiliations:** 1 Centro de Investigación Biomédica en Red Fisiopatología de la Obesidad y la Nutrición (CIBEROBN), Institute of Health Carlos III, Madrid, Spain; 2 Research Group on Nutritional Epidemiology & Cardiovascular Physiopathology. Health Research Institute of the Balearic Islands (IdISBa), University Hospital Son Espases, Balearic Islands, Spain; 3 School of Sport, Exercise and Health Science, Loughborough University, Loughborough, UK; 4 School of Health Sciences, University of Málaga-Institute of Biomedical Research in Malaga (IBIMA), Málaga, Spain; 5 Department of Preventive Medicine and Public Health, IDISNA, University of Navarra, Pamplona, Spain; 6 Department of Nutrition, Harvard T.H. Chan School of Public Health, Boston, MA USA; 7 Universitat Rovira I Virgili, Departament de Bioquímica i Biotecnología, Unitat de Nutrició Humana, Reus, Spain; 8 Institut d’Investigació Sanitària Pere Virgili (IISPV), Hospital Universitari Sant Joan de Reus, Unitat de Nutrició, Reus, Spain; 9 Department of Preventive Medicine, University of Valencia, Valencia, Spain; 10 Unit of Cardiovascular Risk and Nutrition, Institut Hospital del Mar d’Investigacions Mèdiques (IMIM), Barcelona, Spain; 11 CIBER de Epidemiología y Salud Pública (CIBERESP), Instituto de Salud Carlos III, Madrid, Spain; 12 Miguel Hernandez University, ISABIAL-FISABIO, Alicante, Spain; 13 Bioaraba Health Research Institute; Osakidetza Basque Health Service, Araba University Hospital, University of the Basque Country UPV/EHU, Vitoria-Gasteiz, Spain; 14 Department of Nutrition, Food Sciences, and Physiology, Center for Nutrition Research, University of Navarra, Pamplona, Spain; 15 Precision Nutrition and Cardiometabolic Health program, IMDEA Food, CEI UAM + CSIC, Madrid, Spain; 16 Nutrition Research Group, Research Institute of Biomedical and Health Sciences (IUIBS), University of Las Palmas de Gran Canaria, Las Palmas de Gran Canaria, Spain; 17 Department of Internal Medicine, Institut d’Investigacions Biomèdiques August Pi Sunyer (IDIBAPS), Hospital Clinic, University of Barcelona, Barcelona, Spain; 18 Virgen de la Victoria Hospital, Department of Endocrinology, Instituto de Investigación Biomédica de Málaga (IBIMA), University of Málaga, Málaga, Spain; 19 Department of Family Medicine, Research Unit, Distrito Sanitario Atención Primaria Sevilla, Sevilla, Spain; 20 Lipids and Vascular Risk Unit, Internal Medicine, Hospital Universitario de Bellvitge, Hospitalet de Llobregat, Barcelona, Spain; 21 Department of Medicine, Universidad de Barcelona, Barcelona, Spain; 22 Research Group on Community Nutrition & Oxidative Stress, University of Balearic Islands, Palma de Mallorca, Spain; 23 Department of Internal Medicine, Maimonides Biomedical Research Institute of Cordoba (IMIBIC), Reina Sofia University Hospital, University of Cordoba, Cordoba, Spain; 24 Department of Preventive Medicine and Public Health, University of Granada, Granada, Spain; 25 Departamento de Ciencias de la Salud, Centro de Estudios Avanzados en Olivar y Aceites de Oliva, Universidad de Jaén, Jaén, Spain; 26 Department of Endocrinology and Nutrition, Instituto de Investigación Sanitaria Hospital Clínico San Carlos (IdISSC), Madrid, Spain; 27 Nutritional Genomics and Epigenomics Group, IMDEA Food, CEI UAM + CSIC, Madrid, Spain; 28 Cátedrea Cronicidad Universidad Miguel Hernández. Fundación SEMERGEN, Elche, Spain; 29 CIBER Diabetes y Enfermedades Metabólicas (CIBERDEM), Instituto de Salud Carlos III (ISCIII), Madrid, Spain; 30 Department of Endocrinology, Institut d` Investigacions Biomédiques August Pi Sunyer (IDIBAPS), Hospital Clinic, University of Barcelona, Barcelona, Spain; 31 Department of Endocrinology and Nutrition, Hospital Fundación Jimenez Díaz Instituto de Investigaciones Biomédicas IISFJD, University Autonoma, Madrid, Spain; 32 Lipid Clinic, Department of Endocrinology and Nutrition, Institut d’Investigacions Biomèdiques August Pi Sunyer (IDIBAPS), Hospital Clínic, Barcelona, Spain; 33 Emergency Department, Complejo Hospitalario de Navarra, Servicio Navarro de Salud (Osasunbidea), Pamplona, Spain; 34 Public Health Center Son Serra-La Vileta, Primary Care Management, Balearic Islands Health Service, Palma, Spain; 35 Raval Public Health Center, Primary Care Management, Elche, Spain; 36 Department of Clinical Health and Community Sciences (DISCCO), Università degli Studi di Milano, Milan, Italy

**Keywords:** Inactive time, Light physical activity, Moderate to vigorous physical activity, Time in bed, Cardiometabolic risk, Isotemporal substitution

## Abstract

**Background:**

This study explored the association between inactive time and measures of adiposity, clinical parameters, obesity, type 2 diabetes and metabolic syndrome components. It further examined the impact of reallocating inactive time to time in bed, light physical activity (LPA) or moderate-to-vigorous physical activity (MVPA) on cardio-metabolic risk factors, including measures of adiposity and body composition, biochemical parameters and blood pressure in older adults.

**Methods:**

This is a cross-sectional analysis of baseline data from 2189 Caucasian men and women (age 55–75 years, BMI 27–40 Kg/m^2^) from the PREDIMED-Plus study (http://www.predimedplus.com/). All participants had ≥3 components of the metabolic syndrome. Inactive time, physical activity and time in bed were objectively determined using triaxial accelerometers GENEActiv during 7 days (ActivInsights Ltd., Kimbolton, United Kingdom). Multiple adjusted linear and logistic regression models were used. Isotemporal substitution regression modelling was performed to assess the relationship of replacing the amount of time spent in one activity for another, on each outcome, including measures of adiposity and body composition, biochemical parameters and blood pressure in older adults.

**Results:**

Inactive time was associated with indicators of obesity and the metabolic syndrome. Reallocating 30 min per day of inactive time to 30 min per day of time in bed was associated with lower BMI, waist circumference and glycated hemoglobin (HbA1c) (all *p*-values < 0.05). Reallocating 30 min per day of inactive time with 30 min per day of LPA or MVPA was associated with lower BMI, waist circumference, total fat, visceral adipose tissue, HbA1c, glucose, triglycerides, and higher body muscle mass and HDL cholesterol (all p-values < 0.05).

**Conclusions:**

Inactive time was associated with a poor cardio-metabolic profile. Isotemporal substitution of inactive time with MVPA and LPA or time in bed could have beneficial impact on cardio-metabolic health.

**Trial registration:**

The trial was registered at the International Standard Randomized Controlled Trial (ISRCTN: http://www.isrctn.com/ISRCTN89898870) with number 89898870 and registration date of 24 July 2014, retrospectively registered.

## Background

Cardio-metabolic diseases such as type 2 diabetes (T2D) and the metabolic syndrome (MetS) are increasingly prevalent worldwide [1, 2]. Overweight and obesity are major risk factors for these metabolic alterations [3–5] and the World Health Organization (WHO) has projected a significant increase by 2030 [1, 3, 6, 7]. A vast body of the literature suggests that physical activity (PA) and sedentary behaviours, including inactive time and time in bed, are strongly and independently associated with markers of obesity, body composition, and the MetS [2, 8–20]. However, current public health guidelines are mostly focused on the health benefits of moderate-to-vigorous physical activity (MVPA) and less attention is given to inactive time [21, 22].

Research in older adults highlights the health benefits of MVPA and light physical activity (LPA) [2, 4, 23, 24]. However, limited research has explored the associations between time spent inactive, time in bed and cardio-metabolic health in an aging population [4, 13, 23, 25–28]; and, limited research has explored these associations attending the 24-h finite time of a day in a population with chronic conditions.

Isotemporal substitution models have been recommended as one of the most appropriate statistical analysis to explore the associations between reallocating activity patterns, particularly time spent inactive, and health outcomes [4, 13, 23, 26, 28–30]. This type of analyses takes into account that time is finite. Thus, spending time in one behavior (ie. Inactive time) results in less time being spent in another (ie. MVPA) [29], and understands that daily behaviours (sleep, sedentarism and physical activity) are co-dependent [31]. This will provide insightful information that will help better understand the impact of reallocating activity patterns in cardio-metabolic markers in older adults. This is crucial for the design of effective tailored interventions to improve cardiometabolic health in older people in the future. Therefore, this novel study aims to provide new evidence about the associations of inactive time with cardio-metabolic risk factors in an aging population. The outcomes were markers of cardiometabolic health: measures of adiposity and body composition, biochemical parameters, blood pressure, obesity, type 2 diabetes and metabolic syndrome components. The objectives of the present study were a) to explore cross-sectional associations between inactive time and cardio-metabolic risk factors; and b) to assess the impact of replacing 30 min per day of inactive time by 30 min of LPA, MVPA and time in bed on markers of cardio-metabolic health.

## Material and methods

### Study overview and sample

The PREDIMED-Plus study is a 6-year ongoing multicenter, randomized clinical trial, with two intervention arms for the primary prevention of cardiovascular disease in Spain. Details of the study’s protocol have been described elsewhere [32] and are available on the website http://www.predimedplus.com/. Briefly, participant’s in the intervention are receive a multicomponent weight loss intervention that includes an energy-restricted traditional Mediterranean Diet (erMedDiet), PA promotion and behavioural support. Those in the control group receive information about the Mediterranean Diet and cardiovascular health guidelines only. The aim of the study is to prevent cardiovascular disease (a composite of cardiovascular death, non-fatal myocardial infarction, and non-fatal stroke). Eligible participants were men aged 55–75 and women aged 60–75 years, with body mass index (BMI) ≥27 and < 40 kg/m^2^, who met ≥3 components of the MetS [33]. Overall, 6874 men and women were recruited and randomized into the study between 2013 and 2016 across 23 Spanish centers distributed throughout the country’s geography; a subsample of 2260 participants wore an accelerometer at baseline. Participants were asked to wear the accelerometer continuously for at least 7 days. Of those days, we excluded invalid days, i.e. those with less of 10 h of data per day. In addition, we excluded participants with less than 3 days of data [34–36]. Therefore, 2189 participants had valid data, defined as 3 or more days of data with more than 10 h recorded each day. Out of 2189 participants with accelerometer, 662 had additional data on body composition obtained from Dual-energy X-ray absorptiometry (DXA) measurements. All participants provided written informed consent. The study’s protocol was approved by the Research Ethic Committees from all recruiting centers according to the ethical standards of the Declaration of Helsinki. The trial was registered at the International Standard Randomized Controlled Trial (ISRCTN: http://www.isrctn.com/ISRCTN89898870).

### Exposure assessment

Participants were asked to wear an accelerometer on their non-dominant wrist (GENEActiv, ActivInsights Ltd., Kimbolton, United Kingdom) continuously for 7 days. The GENEActiv is a triaxial accelerometer with a dynamic range of ±8 *g*, where *g* is equal to the Earth’s gravitational pull. The GENEActiv was set to capture and store accelerations at a sampling frequency of 40 Hz [37]. As these activity counts are time and date stamped, detailed data on the time, volume, and intensity of movements can be derived [38].

Wrist-worn 3-axial accelerometers do not permit to distinguish standing from sitting or reclining postures, a fact that has conditioned the use of the term inactivity (include all postures) instead of sedentarism (only sitting or reclining postures) in the current study.

Data extracted from the GENEActiv (all in bouts of at least 1 min) was clustered as: inactive time (cut-off intensity level used was < 40 mg) for those behaviours during waking hours equivalent to < 1.5 Metabolic Equivalent Task, METs; LPA (cut-off intensity level used was ≥40 mg and < 100 mg) equivalent to 1.5–3 METs; MVPA (cut-off intensity level used was ≥100 mg) equivalent to > 3 METs; and time in bed (time between going to bed and leaving, calculated using a validated heuristic algorithm from accelerometer raw data unaided by a sleep diary) [37, 39, 40]. For sensitivity analyses we used accelerometer estimated data on sleep time (calculated from accumulated sustained inactivity bouts (SIB) during time in bed, excluding short wake periods (min/night). SIB are detected as the absence of change in arm angle greater than 5 degrees for 5 min or more [41]); nevertheless, our main models are based on time in bed data, given that this estimation has been validated when no information from sleep diaries is available.

Raw data files were managed on servers at the University of Malaga and processed with R-package (R Core Team, Vienna, Austria) using the open-source R-package GGIR, version 1.2–5 (cran.rproject.org/web/packages/GGIR/index.html). This open sources code has been validated in relation to self-calibrated functions [42].

### Outcome assessment

#### Obesity

Obesity prevalence and obesity indicators were determined based on anthropometric parameters. Anthropometric variables were measured by trained personnel according to PREDIMED-Plus protocol [32]. Body weight (kg) and height (cm) were measured in light clothing and without shoes using calibrated scales and a wall-mounted stadiometer. BMI was calculated by dividing the weight (kg) by height in meters squared (m^2^). Obesity was defined as a BMI ≥30 kg/m^2^, and overweight as a BMI ≥ 27 and < 30 kg/m^2^ (given the inclusion criteria, all of our participants had a BMI ≥ 27 and < 40 kg/m^2^). Waist circumference (WC) was measured at the middle point between the last rib and the iliac crest. All anthropometric variables were determined in duplicate, and the average of the two measurements was used.

#### Body composition

Baseline data on total and regional body composition was measured using two types of DXA equipment belonging to the third-generation scanners from GE Healthcare, Madison – WI, connected with EnCore™ software, depending on the availability of this material in the recruiting centers. Total body fat mass (expressed as percentage of total body mass), total body muscle mass (expressed as percentage of total body mass) and Visceral Adipose Tissue (VAT) mass (in kg) were measured. For VAT measures, scans were reanalyzed using validated CoreScan software application [43]. These algorithms work through detection of the width of the subcutaneous tissue layer on the lateral part of the abdomen and the anterior-posterior thickness of the abdomen, by X-ray attenuation of the abdominal cavity in the android region. DXA scans were performed by trained operators following standard protocol and subject positioning provided by the manufacturer. The DXA was phantom calibrated daily according to manufacturer guidelines.

#### Biochemical analyses and clinical determinations

Blood samples were collected after 12 h of overnight fast and biochemical analysis were performed on fasting plasma to determine glucose, glycated hemoglobin (HbA1c), low-density (LDL)-cholesterol, high-density lipoprotein (HDL)-cholesterol and triglycerides concentrations using enzymatic methods. Blood pressure was measured three times with a validated semiautomatic oscillometer (Omron HEM-705CP, the Netherlands) at 5, 10 and 15 min of rest whilst in a seated position.

#### Metabolic syndrome

MetS was defined according to the International Diabetes Federation and the American Heart Association and National Heart, Lung and Blood Institute [33], as having at least 3 of the following components: abdominal obesity for European individuals (WC ≥88 cm in women and ≥ 102 cm in men), hypertriglyceridemia (≥150 mg/dL) or drug treatment for high plasma triglyceride concentration, low HDL (< 50 mg/dL in women and < 40 mg/dL in men), high blood pressure (systolic blood pressure (SBP) ≥130 mmHg or diastolic blood pressure (DBP) ≥85 mmHg) or antihypertensive drug treatment, or high fasting glucose (≥100 mg/dL) or drug treatment for T2D. The presence of MetS was part of the inclusions criteria.

#### Type 2 diabetes

T2D was defined as meeting any of the following criteria: self-reported diabetes at inclusion or baseline, HbA1c ≥ 6.5% or use of antidiabetic medication at baseline, such as insulin, metformin (in case of diagnosed diabetes or Hba1c ≥ 6.5%), and other medication for diabetes.

### Covariate assessment

Baseline data on sex, age, smoking habits, educational level, erMedDiet, marital status, medical conditions and medication use have been evaluated using self-reported questionnaires. Smoking habits was categorized as current, former and never smoker; educational level was categorized as higher education/technician, secondary education and not-completed primary education/primary education; marital status was categorized as married and not married, which included single/separated/divorced/widow (er). Adherence to an energy-restricted Mediterranean diet was measured using a 17-items ErMedDiet score (score range, 0–17; higher scores indicate greater adherence). This score is a modified version of the validated 14-item MEDAS (Mediterranean diet Adherence Screener) used in the PREDIMED study [44]. We also used data on objectively measured muscle strength. Lower-limb muscle strength was determined at baseline using previously validated in community-dwelling older subjects 30s-chair-stand test [45]. This test consists of counting the number of stand-sit on a chair cycles within 30 s. Medication use, including medication for high blood pressure, for high cholesterol, insulin, metformin, and other medications for diabetes treatment, were self-reported by participants at baseline and checked against medical records.

### Statistical analysis

Participants were classified in three categories (tertiles), depending on the inactive time in hours accumulated in one day. Tertile 1 (T1) included those participants accumulating less than 7.6 h/day of inactive time (low time spent with inactive behaviours). Tertile 2 (T2) included those participants who spent between 7.6 and 9.3 h/day inactive (moderate time spent with inactive behaviours). And tertile 3 (T3) included those participants accumulating between 9.3 and 15.1 h/day inactive (high time spent with inactive behaviours).

Descriptive characteristics were summarized as means and standard deviations (SDs) or as numbers and percentages (%). One-way analysis of variance (ANOVA) and Chi-square tests (χ^2^) were used to assess differences across tertiles of inactive time in hours/day for continuous and categorical variables respectively.

First generalized additive models were applied to ascertain about the linearity in the association between our exposures and outcomes. Given that there was no evidence of departure from linearly, multivariate linear regression analyses were used to estimate the β-coefficients and 95% confidence intervals (CIs) for the associations between inactive time (continuous variable: bouts of 30 min; categorical variable: sex-specific tertiles) and BMI, WC, body fat, body muscle mass, VAT, HbA1c, glucose, HDL, LDL, triglycerides, SBP and DBP. Our models were adjusted by the minimally sufficient adjustment set of covariables, determined using Directed Acyclic Graphs (DAGs) implemented in DAGitty software [46] available free on www.dagitty.net. The DAGs were built by identifying all known factors related to inactive time or our outcomes. Therefore our main models were adjusted for age, sex, educational level, marital status, erMedDiet, MVPA and smoking.

Logistic regression models were used to assess the association between categories of inactive time (tertiles) and the prevalence of the MetS’s components, as well as prevalence of obesity and T2D. Prevalence ratios (PR) were calculated using the odds ratios (OR) obtained with logistic regression model. PR permits to assess the true ratios of prevalence in this sample, given the high prevalence of MetS, obesity and T2D in the present population, to avoid an overestimation of the risk. PR were calculated as [(1-P0) + (P0*OR)] (P0 is the prevalence in the reference category) [47].

Linear regression modelling using an isotemporal substitution was used to quantify the associations of replacing 30 min of inactive time for 30 min of time in bed, LPA or MVPA on cardio-metabolic risk markers. Isotemporal substitution has been recommended for use in observational research using time-based measures of physical activity [30]. Prior to running the models, all activity patterns (time in bed, inactive time, LPA and MVPA) were divided by a constant of 30, which was considered as an unit of time equivalent to 30 min (according to the PA guidelines [21, 22, 48]). Consequently, every unit increase represents exchanges of 30 min per day of any of these behaviours. To perform the isotemporal substitution models, a variable representing the total accelerometer wear time was constructed by adding up time in bed, inactive time, LPA and MVPA. This variable of wear time was entered simultaneously in the analysis with time in bed, LPA and MVPA. The resulting regression coefficient represents the association of re-allocating a unit of inactive time to a unit of time in bed, LPA and MVPA. Finally, the model was adjusted for age, sex, educational level, marital status, erMedDiet and smoking. Analyses follow published guideline for isotemporal substitution [31].

Sensitivity analyses were also conducted. Multiple adjusted linear and logistic regression models were adjusted for LPA, instead of MVPA, given that LPA is the most prevalent type of PA in our population and in older adults in general, and some studies had found beneficial effects of LPA on health [4, 5, 26, 27, 49]. In addition, multiple adjusted linear regression models were further adjusted for WC when assessing as an outcome: HbA1c, glucose, HDL, LDL, triglycerides, SBP and DBP. Finally, linear regression models shown in Table 2 were also adjusted for wear time, and results were consistent (data not shown).

In order to test whether the results of the isotemporal replacement models remained similar when using a proxy measure of sleep time, sleep time and time in bed were included in the analysis conjointly with the covariables mentioned above.

Statistical analyses were performed using Stata v15.0 program. *P*-values <0.05 were deemed as statistically significant. All analyses were conducted with data from database PREDIMED-Plus with date 2019-March-12.

## Results

Table 1 presents a comparison of participants’ characteristics among the three categories of inactive time. Participants in the upper tertile (T3) of inactivity were significantly older and had a higher BMI, WC, total body fat, VAT, HbA1c, glucose and triglycerides concentrations, and lower levels of total body muscle mass, and HDL cholesterol. Participants in T3 presented significantly higher prevalence of T2D (38%) and obesity (78%) compared to the other groups, and reported higher consumption of medication for the treatment of diabetes. Those in the most inactive category accumulated the least amount of time in bed, total PA, LPA and MVPA, and accumulated less repeats in the chair-stand test (all *p* values < 0.001). Lastly, the highest prevalence of smokers was found among those in T3 (*p* < 0.001).

**Table 1:** Baseline characteristics of the study population across categories of inactive time measured by accelerometer

Tertiles of inactive time (h/day)^a^						
	Total n	All	T1 *n* = 735	T2 *n* = 729	T3 *n* = 725	*P* - value
Age, years	2189	65.0 (4.95)	64.1 (4.77)	65.2 (4.94)	65.8 (4.98)	< 0.001
Women, n (%)	2189	1032 (47.1)	345 (46.9)	343 (47.1)	344 (47.5)	0.979
Anthropometric measures						
BMI (kg/m^2^)	2189	32.6 (3.46)	32.2 (3.29)	32.4 (3.37)	33.2 (3.63)	< 0.001
Waist circumference (cm)	2189	107 (9.54)	106 (9.41)	107 (9.20)	109 (9.78)	< 0.001
DXA Body composition						
Total body fat (%)	662	40.8 (6.98)	39.4 (6.83)	40.7 (6.84)	42.3 (6.98)	< 0.001
VAT (kg)	651	2.27 (0.90)	2.12 (0.85)	2.32 (0.90)	2.38 (0.93)	0.006
Total body muscle mass (%)	662	56.1 (6.62)	57.4 (6.50)	56.2 (6.50)	54.7 (6.61)	< 0.001
Clinical parameters						
HbA1c (%)	2009	6.15 (0.87)	6.08 (0.81)	6.12 (0.79)	6.26 (0.99)	< 0.001
Glucose (mg/dL)	2164	114 (29.5)	113 (27.9)	113 (27.8)	117 (32.4)	0.035
HDL (mg/dL)	2173	47.8 (11.9)	49.1 (12.0)	47.7 (11.9)	46.6 (11.6)	< 0.001
LDL (mg/dL)	2127	121 (46.5)	122 (31.9)	122 (51.0)	119 (53.7)	0.459
Triglycerides (mg/dL)	2178	152 (78.9)	141 (74.9)	151 (71.4)	165 (87.7)	< 0.001
SBP (mmHg)	2175	140 (17.4)	140 (17.0)	141 (17.3)	140 (18.0)	0.293
DBP (mmHg)	2175	80.3 (10.1)	80.6 (9.88)	80.0 (9.92)	80.2 (10.4)	0.430
Diabetes prevalence, n (%)	2189	749 (34.2)	229 (31.2)	246 (33.7)	274 (37.8)	0.027
Obesity prevalence, n (%)	2189	1610 (73.6)	519 (70.6)	528 (72.4)	563 (77.7)	0.007
Metabolic Syndrome						
High blood pressure, n (%)	2189	1611 (73.6)	534 (72.7)	542 (74.3)	535 (73.8)	0.755
High triglycerides, n (%)	2178	901 (41.4)	244 (33.4)	305 (42.0)	352 (48.9)	< 0.001
Low HDL cholesterol, n (%)	2173	888 (40.9)	263 (36.0)	307 (42.3)	318 (44.3)	0.004
High glucose, n (%)	2189	1686 (77.0)	566 (77.0)	561 (77.0)	559 (77.1)	0.998
High waist circumference, n (%)	2189	2098 (95.8)	706 (96.1)	693 (95.1)	699 (96.4)	0.408
Physical activity behaviours^b^						
Time in bed (h/day)	2189	8.06 (1.29)	8.28 (1.25)	8.23 (1.21)	7.68 (1.33)	< 0.001
Inactive time (h/day)	2189	8.30 (1.98)	6.25 (0.90)	8.21 (0.56)	10.5 (1.31)	< 0.001
LPA (h/day)	2189	2.54 (1.07)	3.45 (0.99)	2.45 (0.74)	1.72 (0.63)	< 0.001
MVPA (h/day)	2187	0.67 (0.54)	0.93 (0.59)	0.66 (0.49)	0.43 (0.40)	< 0.001
Total PA (h/day)	2187	3.22 (1.33)	4.38 (1.22)	3.11 (0.84)	2.15 (0.78)	< 0.001
Chair test 30s (repeats)	2189	13.3 (5.01)	13.8 (5.12)	13.3 (5.00)	12.7 (4.83)	< 0.001
Sociodemographic/lifestyle data						
Smoking habits, n (%)	2181					< 0.001
Never		942 (43.2)	328 (44.8)	329 (45.4)	285 (39.4)	
Current		251 (11.5)	61 (8.3)	75 (10.3)	115 (15.9)	
Former		988 (45.3)	343 (46.9)	321 (44.3)	324 (44.7)	
Educational level, n (%)	2171					< 0.001
Higher education/technician		474 (21.8)	113 (15.5)	176 (24.3)	185 (25.8)	
Secondary education		610 (28.1)	210 (28.8)	198 (27.4)	202 (28.1)	
Primary education/illiterate		1087 (50.1)	406 (55.7)	350 (48.3)	331 (46.1)	
Alcohol intake (g/day)	2187	11.4 (15.4)	11.7 (15.5)	11.0 (14.7)	11.5 (16.1)	0.649
Energy intake (kcal/day)	2187	2407 (633)	2444 (640)	2413 (629)	2364 (628)	0.051
MedDiet score (17 points)	2189	8.56 (2.70)	8.64 (2.75)	8.61 (2.63)	8.42 (2.71)	0.243
Marital status	2179					0.004
Not Married		546 (25.1)	161 (22.0)	174 (23.9)	211 (29.3)	
Married		1633 (74.9)	570 (78.0)	553 (76.1)	510 (70.7)	
Medication use for						
High blood pressure	2189	1700 (77.7)	554 (75.4)	563 (77.2)	583 (80.4)	0.065
Cholesterol	2189	1159 (53.0)	378 (51.4)	384 (52.7)	397 (54.8)	0.437
Insulin	2189	111 (5.07)	36 (4.90)	35 (4.80)	40 (5.52)	0.796
Metformin	2189	544 (24.9)	160 (21.8)	184 (25.2)	200 (27.6)	0.035
Other medications for diabetes	2189	514 (23.5)	151 (20.5)	171 (23.5)	192 (26.5)	0.028

Data shown is mean (SD), unless otherwise specified; Abbreviations: *BMI* body mass index, *VAT* visceral adipose tissue, *SBP* systolic blood pressure, *DBP* diastolic blood pressure, *LDL* low density lipoproteins, *HDL* high density lipoproteins, *HbA1c* glycated haemoglobin, *PA* physical activity, *LPA* light physical activity, *MVPA* moderate-to-vigorous physical activity, *MedDiet* Mediterranean diet. ^a^ T1, Men (min 3.79 / max 7.6), Women (min 2.64 / max 7.15). T2 Men (min 7.61 / 9.34max); Women (min 7.16 / 8.73max). T3, Men (min 9.35 / 17.2 max), Women (min 8.74/ 15.15max). Tertiles were calculated using the total sample of 2189, and the sample size shown corresponds to the distribution of these 2189 individuals within tertiles; the sample size within tertiles varied for outcome variables with different total sample size. Sample sizes in tertiles of body composition variables determined by DXA were: T1, *n* = 225; T2, *n* = 219; T3, *n* = 218. ^b^Data shown of time in bed, physical activity and inactive time has been recorded by accelerometer. High waist circumference was defined has a circumference ≥ 120 cm in men and ≥ 88 cm in women. High glucose was defined as ≥110 mg/dl of glucose in blood or antidiabetic treatment with metformin or insulin. High levels of triglycerides was defined as ≥150 mg/dl of blood. Low levels of HDL cholesterol was defined as ≤40 mg/dl in men and ≤ 50 mg/dl in women. Obesity was defined with a BMI ≥30 kg/m^2^. Hypertension was defined with a SBP ≥90 mmHg and a SBP ≥140 mmHg. It was considered diabetes if the participant has a previous diagnosis of diabetes, with a HbA1c (%) ≥6.5, or with the use of medication for diabetes treatment as insulin and metformin

Table 2 shows the β-coefficients (95% CIs) for the associations between total inactive time, (both per 30-min bouts and in tertiles) and anthropometric measurements, body composition, biochemical parameters and blood pressure. Higher inactive time was associated with a worse adiposity and cardio-metabolic profile, including statistically significant higher BMI, WC, total body fat, VAT, HbAc1, glucose, triglycerides and DBP, and lower total body muscle mass and HDL cholesterol level.

**Table 2:** Associations of total inactive time with adiposity indicators and cardio-metabolic risk factors

	Tertiles of inactive time (h/day)^a^			*p for trend*	Continuous (per 30 min/d of inactive time)	*p-value*
Outcome	T1 *n* = 735	T2 *n* = 729	T3 *n* = 725			
Anthropometric measures						
BMI (kg/m^2^)	0 (ref.)	0.22 (−0.14;0.58)	0.90 (0.52;1.29)	< 0.001	0.11 (0.06;0.15)	< 0.001
Waist circumference (cm)	0 (ref.)	−0.05 (− 0.99;0.89)	1.97 (0.96;2.97)	< 0.001	0.26 (0.15;0.37)	< 0.001
Body Composition						
Total body fat (%)	0 (ref.)	0.69 (−0.14;1.53)	1.41 (0.50;2.32)	0.002	0.17 (0.07;0.27)	0.001
VAT (Kg)	0 (ref.)	0.16 (0.00;0.31)	0.23 (0.06;0.39)	0.013	0.03 (0.01;0.05)	0.002
Total body muscle mass (%)	0 (ref.)	−0.66 (−1.46;0.14)	−1.30 (−2.17;-0.44)	0.003	− 0.16 (− 0.25;-0.06)	0.002
Clinical Parameters						
HbA1c (%)	0 (ref.)	0.01 (−0.09;0.10)	0.11 (0.01;0.21)	0.032	0.02 (0.01;0.03)	< 0.001
Glucose (mg/dL)	0 (ref.)	0.08 (−3.06;3.22)	2.68 (−0.68;6.05)	0.116	0.47 (0.11;0.83)	0.010
HDL (mg/dL)	0 (ref.)	−1.02 (−2.19;0.15)	− 1.64 (− 2.89;-0.39)	0.010	−0.20 (− 0.34;-0.07)	0.003
LDL (mg/dL)	0 (ref.)	1.16 (−3.87;6.19)	− 1.23 (−6.63;4.17)	0.652	−0.19 (− 0.76;0.39)	0.526
Triglycerides (mg/dL)	0 (ref.)	8.58 (0.25;16.9)	20.0 (11.0;28.9)	< 0.001	2.15 (1.20;3.11)	< 0.001
SBP (mmHg)	0 (ref.)	1.43 (−0.41;3.27)	0.34 (−1.63;2.32)	0.740	−0.00 (− 0.21;0.21)	0.981
DBP (mmHg)	0 (ref.)	0.02 (−1.03;1.06)	0.74 (−0.38;1.86)	0.195	0.14 (0.02;0.26)	0.022

Values shown are β (95% CI). Abbreviations: *BMI* body mass index, *VAT* visceral adipose tissue, *HbA1c* glycated haemoglobin, *HDL* high density lipoprotein, *LDL* low density lipoprotein, *SBP* systolic blood pressure, *DBP*; diastolic blood pressure. Linear regression models were used to assess the association between inactive time and each cardiometabolic risk outcomes, adjusting for age, sex, education level, marital status, erMedDiet, moderate-vigorous physical activity and smoking. ^a^ T1, Men (min 3.79 / max 7.6), Women (min 2.64 / max 7.15). T2 Men (min 7.61 / 9.34max); Women (min 7.16 / 8.73max). T3, Men (min 9.35 / 17.2 max), Women (min 8.74/ 15.15max). Tertiles were calculated using the total sample of 2189, and the sample size shown corresponds to the distribution of these 2189 individuals within tertiles; the sample size within tertiles varied for outcome variables with different total sample size. Sample sizes in tertiles of body composition variables determined by DXA were: T1, *n* = 225; T2, *n* = 219; T3, *n* = 218

Table 3 shows the prevalence ratios for obesity, T2D and the MetS’s components by categories of inactive time. Those in the most inactive category (T3) showed significantly higher obesity prevalence (p for trend = 0.014), significantly higher triglycerides (*p* = 0.005), and higher number of MetS components, ≥4 components (p for tend = 0.051) and 5 components (p for tend = 0.054), compared to those with less inactive time (T1).

**Table 3:** Prevalence Ratio of clinical and metabolic syndrome parameters according to tertiles of inactive time

	Tertiles of inactive time (h/day)^a^			*p for trend*
Outcome	T1 *n* = 735	T2 *n* = 729	T3 *n* = 725	
Obesity prevalence	1 (ref.)	1.02 (0.95;1.09)	1.09 (1.02;1.15)	0.014
Diabetes prevalence	1 (ref.)	1.05 (0.89;1.21)	1.14 (0.96;1.32)	0.125
Metabolic Syndrome Components				
High blood pressure	1 (ref.)	0.97 (0.93;1.00)	1.01 (0.98;1.03)	0.580
High triglycerides	1 (ref.)	1.11 (1.01;1.21)	1.16 (1.05;1.26)	0.005
Low HDL cholesterol	1 (ref.)	1.08 (0.96;1.22)	1.05 (0.90;1.19)	0.533
High glucose	1 (ref.)	0.99 (0.93;1.05)	0.99 (0.92;1.05)	0.674
High waist circumference	1 (ref.)	0.99 (0.96;1.01)	1.00 (0.97;1.02)	0.954
≥ 4 components metabolic syndrome	1 (ref.)	1.09 (0.97;1.21)	1.13 (1.00;1.25)	0.051
5 components metabolic syndrome	1 (ref.)	1.08 (0.82;1.40)	1.30 (0.99;1.67)	0.054

Values shown are prevalence ratios (95% CI). Abbreviations: *HDL* high-density lipoprotein. Logistic regression models were adjusted for age, sex, educational level, marital status, erMedDiet, moderate-vigorous physical activity and smoking status. Tertiles were calculated using the total sample of 2189, and the sample size shown corresponds to the distribution of these 2189 individuals within tertiles; the sample size within tertiles varied for outcome variables with different total sample size

^a^T1, Men (min 3.79 / max 7.6), Women (min 2.64 / max 7.15). T2 Men (min 7.61 / 9.34max); Women (min 7.16 / 8.73max). T3, Men (min 9.35 / 17.2 max), Women (min 8.74/ 15.15max). Tertiles were calculated using the total sample of 2189, and the sample size shown corresponds to the distribution of these 2189 individuals within tertiles.

**Fig. 1:**
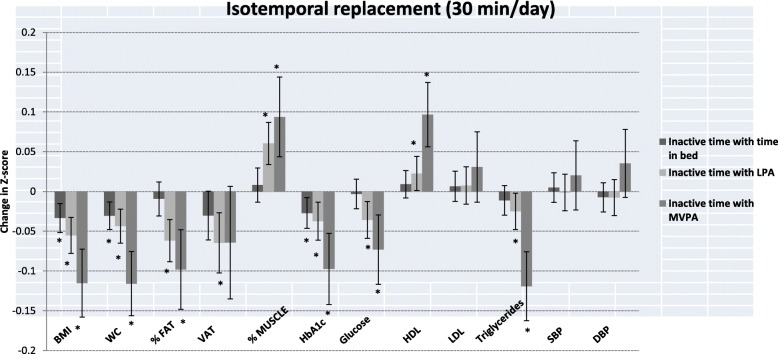
Isotemporal substitution of inactive time (30 min/day) with time in bed and physical activity on standardized cardio-metabolic risk. Values shown are β (95% CI). These represent the change in outcome variables (z-scores) when substituting 30 min per day of inactive time with time in bed and physical activity. Abbreviations: LPA: light physical activity; MVPA: moderate-vigorous physical activity; BMI: body mass index; VAT: visceral adipose tissue; HbA1c: glycated haemoglobin; HDL: high density lipoprotein; LDL: low-density lipoprotein; SBP: systolic blood pressure; DBP: diastolic blood pressure. Linear regression models were used to assess isotemporal substitution of inactive time with time in bed, light PA and MVPA, adjusting for age, sex, educational level, marital status, erMedDiet, and smoking.*indicates *p* value < 0.05

Table 4 shows the β-coefficients (95% CIs) of the isotemporal substitution models. Figure 1 shows the same isotemporal substitution models but outcome variables had been standardized as z-scores to aid visualization of results. Isotemporal substitution of 30 min a day of inactive time with equivalent time in bed was associated with lower BMI, WC and HbA1c (all *p*-values < 0.05); reallocating 30 min of inactive time per day with LPA or MVPA (i.e., decreasing inactive time at the expense of increasing LPA or MVPA time) was associated with lower BMI, WC, total body fat, VAT, HbA1c, glucose, triglycerides, and higher total body muscle mass and HDL (all p-values < 0.05). Estimates of association were larger in all variables when replacing 30 min a day of inactive time by the equal amount of time in MVPA than when replacing it by LPA or time in bed.

**Table 4:** Isotemporal substitution of inactive time (30 min/day) with time in bed and physical activity on cardio-metabolic risk

Outcome	Inactive time with time in bed	*p-value*	Inactive time with LPA	*p-value*	Inactive time with MVPA	*p-value*
Anthropometric measures						
BMI (kg/m^2^)	−0.12 (−0.18;-0.05)	< 0.001	−0.19 (− 0.27;-0.11)	< 0.001	− 0.40 (− 0.55;-0.25)	< 0.001
Waist circumference (cm)	− 0.29 (− 0.46;-0.13)	0.001	−0.42 (− 0.62;-0.21)	< 0.001	−1.11 (−1.49;-0.72)	< 0.001
Body composition						
Total body fat (%)	−0.07 (− 0.21;0.08)	0.391	−0.43 (− 0.62;-0.25)	< 0.001	−0.69 (− 1.03;-0.34)	< 0.001
VAT (Kg)	− 0.03 (− 0.05;0.00)	0.052	−0.06 (− 0.09;-0.03)	0.001	−0.06 (− 0.12;0.01)	0.075
Total body muscle mass (%)	0.05 (−0.09;0.20)	0.465	0.40 (0.22;0.56)	< 0.001	0.62 (0.29;0.95)	< 0.001
Clinical parameters						
HbA1c (%)	−0.02 (− 0.04;-0.01)	0.006	− 0.03 (− 0.05;-0.01)	0.002	−0.08 (− 0.12;-0.05)	< 0.001
Glucose (mg/dL)	− 0.09 (− 0.64;0.45)	0.736	−1.05 (− 1.73;-0.38)	0.002	−2.15 (−3.44;-0.87)	0.001
HDL (mg/dL)	0.11 (− 0.10;0.31)	0.306	0.27 (0.01;0.52)	0.039	1.15 (0.67;1.63)	< 0.001
LDL (mg/dL)	0.30 (−0.58;1.19)	0.502	0.34 (−0.75;1.43)	0.538	1.43 (−0.63;3.48)	0.174
Triglycerides (mg/dL)	−0.89 (−2.35;0.58)	0.235	−1.98 (−3.78;-0.17)	0.032	−9.40 (−12.8;-5.99)	< 0.001
SBP (mmHg)	0.09 (−0.24;0.41)	0.597	−0.02 (− 0.42;0.38)	0.906	0.35 (− 0.41;1.11)	0.363
DBP (mmHg)	−0.07 (− 0.26;0.11)	0.431	− 0.08 (− 0.30;0.15)	0.505	0.36 (− 0.07;0.79)	0.105

Values shown are β (95% CI). These represent the change in outcome variables when substituting 30 min/day of inactive time with time in bed and physical activity. Abbreviations: *LPA* light physical activity, *MVPA* moderate-vigorous physical activity, *BMI* body mass index, *VAT* visceral adipose tissue, *HbA1c* glycated haemoglobin, *HDL* high density lipoprotein, *LDL* low-density lipoprotein, *SBP*; systolic blood pressure, *DBP* diastolic blood pressure. Linear regression models were used to assess isotemporal substitution of inactive time with: time in bed, light PA and MVPA, adjusting for age, sex, educational level, marital status, erMedDiet, smoking and total wear time

^a^T1, Men (min 3.79 / max 7.6), Women (min 2.64 / max 7.15). T2 Men (min 7.61 / 9.34max); Women (min 7.16 / 8.73max). T3, Men (min 9.35 / 17.2 max), Women (min 8.74/ 15.15max). Tertiles were calculated using the total sample of 2189, and the sample size shown corresponds to the distribution of these 2189 individuals within tertiles

No significant changes were observed when performing sensitivity analyses adjusting linear and logistic regression models for LPA instead of MVPA (See Additional file 1: Table S1 and Table S3), or for WC (See Additional file 1: Table S2). When running the isotemporal replacement models with sleep time instead of time in bed results also remained similar (See Additional file 1: Table S4).

## Discussion

Results from this cross-sectional study show that time spent inactive was associated with a number of cardio-metabolic risk factors in a sample of older adults, independent of PA levels. Overall, this study highlights that replacing 30 min a day of inactive time with an equal amount of MVPA, LPA and time in bed resulted in a significantly improved cardio-metabolic profile in men and women with the MetS.

The results from this study show that inactive time worsens the metabolic profile in an aging population with high cardio-metabolic risk, increasing the chances of cardiovascular events. This is similar to other studies where it has been found that high levels of inactivity, including sitting time, are associated with higher rates of obesity, triglycerides and MetS [50, 51] and premature mortality and diabetes [51, 52] across different populations.

Isotemporal substitution analyses have public health implications [4, 9, 13, 23, 25, 28, 29, 31]. Comparative research in older adults is limited, especially in a population with chronic conditions, such as MetS [25]. Thus, the present results are in line with previous research conducted in adults (18–79 years) [4, 9, 13, 23, 25], which shows the beneficial effects of exchanging an unit of time spent inactive with equal amounts of PA or sleep on cardio-metabolic risk factors, including obesity and lipid profile. This study shows that replacing inactive time with any other behaviors has beneficial effects on cardio-metabolic risk, and these benefits increase proportionally. For instance, replacing 30 min/d of inactive time with 30 min/d of time in bed was associated with a lower WC of − 0.26 cm, whereas replacing this amount of inactive time with LPA resulted in a WC of − 0.45 cm and with MVPA in a WC of − 1.08 cm.

Previous studies in adults have highlighted the benefits of replacing inactive time with MVPA, with the greatest benefits on improved BMI [9], T2D [9, 23], triglycerides, HbA1c [23] and glucose. Similarly, this study shows that interchanging 30 min of time inactive by MVPA was significantly associated with improvements in BMI, WC, body fat, muscle mass, HbA1c, glucose, HDL and triglycerides. Furthermore, the present study shows that health benefits are also attained when time inactive is replaced by LPA or time in bed, with improvements on: BMI, WC, body fat, VAT, muscle mass, HbA1c, glucose, HDL and triglycerides (LPA); and BMI, WC and HbA1c (time in bed). This is of interest, as research on this area continuous to mount, however findings remain ambiguous [4, 9, 10, 13, 23–25, 23] and although there are some studies in adult population [4, 25, 28, 29], few research has been conducted in older adults [23, 26] and, as far as we know, none in individuals with overweight/obesity and metabolic syndrome.

Given the prevalence of MetS, and the prevalence of an population aging worldwide, effective and sustainable long term actions are needed. Understanding the beneficial effects of substituting time inactive with different activity levels and sleep in high risk and aging populations is of importance as it will help defining future tailored health interventions. Multicomponent interventions to increase PA and decrease inactive time, using a multidisciplinary approach are recommended. According to our results, the promotion of MVPA would be of most benefit, however in older adults designing health interventions focused on LPA and sleep might be more appropriate. Interventions focused on LPA and sleep might result more feasible, appealing and might help improve attrition and sustainability in the long term, as they will not need continuous supervision and are easy to implement at home or care homes.

A marked strength of this study was the use of a large cohort of older men and women, with overweight/obesity and MetS. It is important to highlight that only objective and validated measurements were used for this study for both exposure and outcome variables. This reduces any potential bias or measurement error and increases the opportunities for comparison across the literature. In terms of limitations, the cross-sectional design prevents the assessment of causality. Given that exposure and outcome variables were measured simultaneously, we cannot rule out reverse causation, i.e. our outcomes, such as obesity, may have preceded inactivity, and not the other way round. In addition, due to its cross-sectional study design, the isotemporal replacement model used in this study is not based on actual replacements of one activity for another and should be interpreted at the population level; longitudinal studies are necessary to confirm the results obtained in this study. Selection of older subjects with overweight/obesity and MetS for the study cohort limits extrapolation of findings to other populations, including younger, leaner or healthier subjects. Moreover, this study was limited to Caucasians, hence the associations found may not be applicable to other ethnic groups. Thus, replicating this research in different ethnic groups with different lifestyles and fat distribution would be of interest. Methodological limitations of differentiating between sitting, standing are also important to consider. The wrist-worn 3-axial accelerometers used in this study quantify time spent in different intensities of activity based on specific count thresholds. This method works reasonably well for identifying inactive, LPA and MVPA but it is limited in its capacity to distinguish between standing and reclining postures. Thus, throughout this paper we refer to “inactivity” (activities of < 1.5 METs during day-time) and not “sedentarism” (meaning activities of < 1.5 METs/day in seated or reclining positions). Another limitation is the use of the cutoff intensity level points to cluster data as inactive, LPA or MVPA time. Cut points are normally population and protocol specific, limiting the possibility of comparison across studies and populations [35]. Finally, although we used validated algorithms to estimate time in bed from accelerometer data without the use of sleep diaries, sleep time estimates were less accurate, which prevented us from using sleep time in the main analyses. This issue has been overcame by using several sophisticated analysis to assess the complex inter-relationships between different lifestyle behaviours in relation to cardio-metabolic risk factors.

## Conclusion

These results add to the growing literature using Isotemporal Replacement methods and it is one of the few focused on older adults with the metabolic syndrome. Results from this cross-sectional study indicate that replacing inactive time with any PA and time in bed was associated with improved cardio-metabolic factors in older adults with overweight or obesity and the MetS. Our findings support the notion that PA and inactive time are both linked with health outcomes and that both behaviors should be included in public health guidelines. Future intervention studies are needed to confirm causality. Tailored health intervention research with a focus on sleep, LPA and MVPA are recommended.

## Supplementary information

**Additional file 1: Table S1.** Associations of total inactive time with adiposity indicators and cardio-metabolic risk factors (with further adjustment for light physical activity, instead of moderate-to-vigorous physical activity). **Table S2.** Associations of total inactive time with adiposity indicators and cardio-metabolic risk factors (with further adjustment for light physical activity, instead of moderate-to-vigorous physical activity and waist circumference as an indicator of adiposity). **Table S3.** Prevalence ratio of metabolic syndrome parameters according to tertiles of sedentary time (with further adjustment for light physical activity, instead of moderate-to-vigorous physical activity). **Table S4.** Isotemporal substitution of inactive time (30 min/day) with sleep time and physical activity on cardio-metabolic risk.

## Data Availability

There are restrictions on the availability of data for the PREDIMED-Plus trial, due to the signed consent agreements around data sharing, which only allow access to external researchers for studies following the project purposes. Requestors wishing to access the PREDIMED-Plus trial data used in this study can make a request to the PREDIMED-Plus trial Steering Committee chair: jordi.salas@urv.cat. The request will then be passed to members of the PREDIMED-Plus Steering Committee for deliberation.

## Abbreviations

BMI: Body mass index
CIs: Confidence intervals
DAG: Directed acyclic graphs
DBP: Diastolic blood pressure
DXA: Dual-energy X-ray absorptiometry
erMedDiet: Energy-restricted traditional Mediterranean Diet
HbA1c: Glycated hemoglobin
HDL: High-density lipoprotein cholesterol
LDL: Low-density lipoprotein cholesterol
LPA: Light physical activity
METs: Metabolic equivalent tasks
MetS: Metabolic syndrome
MVPA: Moderate-to-vigorous physical activity
OR: Odds ratios
PA: Physical activity
PR: Prevalence ratios
SBP: Systolic blood pressure
SDs: Standard deviations
T2D: Type 2 diabetes
VAT: Visceral adipose tissue
WC: Waist circumference
WHO: World health organization
