# Supplementary material for: Isotemporal substitution of inactive time with physical activity and time in bed: cross-sectional associations with cardiometabolic health in the PREDIMED-Plus study
Source: Int J Behav Nutr Phys Act. 2019 Dec 23;16:137. doi: 10.1186/s12966-019-0892-4 (PMC6929461; doi:10.1186/s12966-019-0892-4)
Supplement: Supplementary file 1 — Additional file 1: Table S1. Associations of total inactive time with adiposity indicators and cardio-metabolic risk factors (with further adjustment for light physical activity, instead of moderate-to-vigorous physical activity). Table S2. Associations of total inactive time with adiposity indicators and cardio-metabolic risk factors (with further adjustment for light physical activity, instead of moderate-to-vigorous physical activity and waist circumference as an indicator of adiposity). Table S3. Prevalence ratio of metabolic syndrome parameters according to tertiles of sedentary time (with further adjustment for light physical activity, instead of moderate-to-vigorous physical activity). Table S4. Isotemporal substitution of inactive time (30 min/day) with sleep time and physical activity on cardio-metabolic risk. [file 12966_2019_892_MOESM1_ESM.docx]

| **Table S1.** Associations of total inactive time with adiposity indicators and cardio-metabolic risk factors (with further adjustment for light physical activity, instead of moderate-to-vigorous physical activity) | | | | | | |
| --- | --- | --- | --- | --- | --- | --- |
|  | Tertiles of sedentarism (h/day) | | | *p for trend* | Continuous (per 30 min/d) | *p-value* |
| Outcome | T1  n=735 | T2  n=729 | T3  n=725 |  |  |  |
| **Anthropometric measures** |  |  |  |  |  |  |
| BMI (kg/m^2^) | ref | 0.16 (-0.24;0.56) | 0.83 (0.36;1.31) | <0.001 | 0.11 (0.06;0.16) | <0.001 |
| Waist circumference (cm) | ref | -0.14 (-1.17;0.90) | 1.88 (0.64;3.12) | 0.002 | 0.31 (0.17;0.45) | <0.001 |
| **Body composition** |  |  |  |  |  |  |
| Total body fat (%) | ref | 0.46 (-0.43;1.35) | 0.89 (-0.18;1.97) | 0.103 | 0.11 (-0.02;0.24) | 0.096 |
| VAT (Kg) | ref | 0.12 (-0.04;0.29) | 0.16 (-0.03;0.36) | 0.082 | 0.02 (-0.00;0.05) | 0.055 |
| Total body muscle mass (%) | ref | -0.43 (-1.27;0.42) | -0.81 (-1.83;0.22) | 0.122 | -0.09 (-0.22;0.03) | 0.125 |
| **Clinical parameters** |  |  |  |  |  |  |
| HbA1c (%) | ref | -0.02 (-0.12;0.09) | 0.08 (-0.05;0.20) | 0.205 | 0.03 (0.01;0.04) | <0.001 |
| Glucose (mg/dL) | ref | -1.46 (-4.91;1.99) | 0.18 (-3.96;4.32) | 0.883 | 0.32 (-0.14;0.78) | 0.178 |
| HDL (mg/dL) | ref | -0.97 (-2.25;0.32) | -1.64 (-3.19;-0.09) | 0.039 | -0.24 (-0.41;-0.06) | 0.007 |
| LDL (mg/dL) | ref | 0.81 (-4.71;6.33) | -1.86 (-8.51;4.79) | 0.556 | -0.32 (-1.06;0.42) | 0.397 |
| Triglycerides (mg/dL) | ref | 8.57 (-0.61;17.8) | 20.6 (9.58;31.7) | <0.001 | 2.41 (1.18;3.64) | <0.001 |
| SBP (mmHg) | ref | 1.31 (-0.72;3.33) | 0.09 (-2.34;2.52) | 0.993 | -0.08 (-0.35;0.19) | 0.581 |
| DBP (mmHg) | ref | -0.40 (-1.55;0.74) | -0.02 (-1.39;1.36) | 0.980 | 0.09 (-0.07;0.24) | 0.276 |

*Values shown are β (95% CI)

Abbreviations: BMI; body mass index, VAT; visceral adipose tissue, HbA1c; glycated haemoglobin, HDL; high-density lipoprotein, LDL; low-density lipoprotein, SBP; systolic blood pressure, DBP; diastolic blood pressure. Tertiles were calculated using the total sample of 2189, and the sample size shown corresponds to the distribution of these 2189 individuals within tertiles; the sample size within tertiles varied for outcome variables with different total sample size. Sample sizes in tertiles of body composition variables determined by DXA were: T1, n=225; T2, n=219; T3, n=218.

Linear regression models were used to assess the association between inactive time (per 30 min/day increments) and each cardio-metabolic risk outcomes, adjusting for age, sex, educational level, marital status, erMedDiet, light physical activity, and smoking.

| **Table S2.** Associations of total inactive time with adiposity indicators and cardio-metabolic risk factors (with further adjustment for light physical activity, instead of moderate-to-vigorous physical activity and waist circumference as an indicator of adiposity) | | | | | | |
| --- | --- | --- | --- | --- | --- | --- |
|  | Tertiles of sedentarism (h/day) | | | *p for trend* | Continuous (per 30 min/d) | *p-value* |
| Outcome | T1  n=735 | T2  n=729 | T3  n=725 |  |  |  |
| HbA1c (%) | ref | -0.01 (-0.12;0.09) | 0.06 (-0.07;0.18) | 0.354 | 0.02 (0.01;0.04) | 0.001 |
| Glucose (mg/dL) | ref | -1.39 (-4.80;2.02) | -0.80 (-4.90;3.31) | 0.731 | 0.16 (-0.30;0.61) | 0.501 |
| HDL (mg/dL) | ref | -0.98 (-2.27;0.30) | -1.46 (-3.01;0.09) | 0.067 | -0.21 (-0.38;-0.04) | 0.018 |
| LDL (mg/dL) | ref | 0.81 (-4.71;6.33) | -1.86 (-8.53;4.80) | 0.559 | -0.32 (-1.06;0.42) | 0.398 |
| Triglycerides (mg/dL) | ref | 8.69 (-0.47;17.8) | 19.4 (8.36;30.4) | 0.001 | 2.21 (0.98;3.44) | <0.001 |
| SBP (mmHg) | ref | 1.31 (-0.71;3.33) | -0.00 (-2.44;2.43) | 0.933 | -0.09 (-0.36;0.18) | 0.506 |
| DBP (mmHg) | ref | -0.40 (-1.55;0.74) | -0.02 (-1.40;1.37) | 0.983 | 0.09 (-0.07;0.24) | 0.276 |

*Values shown are β (95% CI)

Abbreviations: HbA1c; glycated haemoglobin, HDL; high-density lipoprotein, LDL; low-density lipoprotein, SBP; systolic blood pressure, DBP; diastolic blood pressure. Tertiles were calculated using the total sample of 2189, and the sample size shown corresponds to the distribution of these 2189 individuals within tertiles; the sample size within tertiles varied for outcome variables with different total sample size.

Linear regression models were used to assess the association between total sedentary time (per 30 min/day increments) and each cardiometabolic risk outcomes, adjusted for age, sex, educational level, marital status, erMedDiet, light physical activity, smoking and waist circumference.

| **Table S3.** Prevalence ratio of metabolic syndrome parameters according to tertiles of sedentary time (with further adjustment for light physical activity, instead of moderate-to-vigorous physical activity) | | | | |
| --- | --- | --- | --- | --- |
|  | Categories of sedentary time in tertiles | | | *p for trend* |
| Outcome | T1  n=735 | T2  n=729 | T3  n=725 |  |
| Obesity prevalence | ref | 1.04 (0.97;1.11) | 1.12 (1.03;1.18) | 0.007 |
| Diabetes prevalence | ref | 0.96(0.80;1.14) | 1.00 (0.80;1.22) | 0.971 |
| **Metabolic Syndrome Components** |  |  |  |  |
| High blood pressure | ref | 0.97 (0.92;1.00) | 1.00 (0.95;1.03) | 0.992 |
| High triglycerides | ref | 1.08 (0.97;1.19) | 1.11 (0.97;1.24) | 0.125 |
| Low HDL cholesterol | ref | 1.10 (0.96;1.25) | 1.08 (0.91;1.26) | 0.387 |
| High glucose | ref | 0.98 (0.91;1.04) | 0.97 (0.88;1.04) | 0.420 |
| High waist circumference | ref | 0.99 (0.94;1.01) | 1.00 (0.95;1.02) | 0.934 |
| ≥ 4 components metabolic syndrome | ref | 1.04 (0.91;1.17) | 1.05 (0.90;1.21) | 0.510 |
| 5 components metabolic syndrome | ref | 1.04 (0.78;1.39) | 1.24 (0.88;1.71) | 0.178 |

Values shown are β (95% CI)

Abbreviations: HDL; high-density lipoprotein.

Tertiles were calculated using the total sample of 2189, and the sample size shown corresponds to the distribution of these 2189 individuals within tertiles; the sample size within tertiles varied for outcome variables with different total sample size.

All variables were adjusted according to model 2: age, sex, educational level, marital status, erMedDiet, light physical activity and smoking status.

| **Table S4**. Isotemporal substitution of inactive time (30 min/day) with sleep time and physical activity on cardio-metabolic risk | | | | | | | |
| --- | --- | --- | --- | --- | --- | --- | --- |
| Outcome | Inactive time with sleep time | *p-value* | Inactive time with LPA | *p-value* | Inactive time with MVPA | *p-value* |  |
| **Anthropometric measures** |  |  |  |  |  |  |  |
| BMI (kg/m^2^) | -0.16 (-0.23;-0.10) | <0.001 | -0.19 (-0.26;-0.11) | <0.001 | -0.36 (-0.51;-0.21) | <0.001 |  |
| Waist circumference (cm) | -0.40 (-0.57;-0.23) | <0.001 | -0.41 (-0.61;-0.20) | <0.001 | -1.02 (-1.41;-0.64) | <0.001 |  |
| **Body composition** |  |  |  |  |  |  |  |
| Total body fat (%) | -0.09 (-0.24;0.06) | 0.232 | -0.42 (-0.61;-0.24) | <0.001 | -0.67 (-1.02;-0.32) | <0.001 |  |
| VAT (Kg) | -0.04 (-0.06;-0.01) | 0.013 | -0.06 (-0.09;-0.02) | 0.001 | -0.05 (-0.12;0.01) | 0.091 |  |
| Total body muscle mass (%) | 0.08 (-0.07;0.22) | 0.295 | 0.39 (0.21;0.57) | <0.001 | 0.61 (0.28;0.94) | <0.001 |  |
| **Clinical parameters** |  |  |  |  |  |  |  |
| HbA1c (%) | -0.03 (-0.05;-0.01) | 0.001 | -0.03 (-0.05;-0.01) | 0.003 | -0.08 (-0.12;-0.04) | <0.001 |  |
| Glucose (mg/dL) | -0.30 (-0.86;0.27) | 0.306 | -1.04 (-1.72;-0.36) | 0.003 | -1.98 (-3.27;-0.69) | 0.003 |  |
| HDL (mg/dL) | 0.14 (-0.08;0.35) | 0.212 | 0.26 (0.01;0.52) | 0.041 | 1.12 (0.64;1.61) | <0.001 |  |
| LDL (mg/dL) | 0.19 (-0.72;1.11) | 0.678 | 0.35 (-0.74;1.44) | 0.526 | 1.51 (-0.55;3.57) | 0.151 |  |
| Triglycerides (mg/dL) | -0.88 (-2.39;0.64) | 0.255 | -1.98 (-3.79;-0.17) | 0.032 | -9.41 (-12.8;-5.98) | <0.001 |  |
| SBP (mmHg) | 0.01 (-0.33;0.34) | 0.965 | -0.02 (-0.42;0.38) | 0.932 | 0.42 (-0.35;1.18) | 0.284 |  |
| DBP (mmHg) | -0.13 (-0.32;0.06) | 0.178 | -0.07 (-0.30;0.15) | 0.532 | 0.40 (-0.03;0.83) | 0.069 |  |

Values shown are β (95% CI). These represent the change in outcome variables when substituting 30 min/day of inactive time with sleep time and physical activity. Abbreviations: LPA; light physical activity, MVPA; moderate-vigorous physical activity, BMI; body mass index, VAT; visceral adipose tissue, HbA1c; glycated haemoglobin, HDL; high density lipoprotein, LDL; low-density lipoprotein, SBP; systolic blood pressure, DBP; diastolic blood pressure. Linear regression models were used to assess isotemporal substitution of inactive time with: sleep time, light PA and MVPA, adjusting for age, sex, educational level, marital status, erMedDiet, and smoking.
